# Supplementary material for: Returning to Leisure Activity Post-Stroke: Barriers and Facilitators to Engagement
Source: Int J Environ Res Public Health. 2022 Nov 7;19(21):14587. doi: 10.3390/ijerph192114587 (PMC9657985; doi:10.3390/ijerph192114587)
Supplement: Supplementary file 1 [file ijerph-19-14587-s001.zip › ijerph-1966945-supplementary.pdf]

Supplementary Information S1. Number of participant characteristics identified within the 'barrier' themes as a proportion (%) of the total participant characteristics identified for responses to the 'made it more difficult' question

Participant characteristics	Physical difficulties (n=717)		Energy Levels (n=179)		Losing in-dependence (n=111)		Mental health difficulties (n=109)		Hidden Disability (n=69)		Delay or lack of healthcare provision (n=31)		'made it more difficult' response total (n=1045)
**Event**													
Stroke	612	(70)	149	(17)	101	(12)	89	(10)	55	(6)	26	(3)	872
TIA	105	(61)	30	(17)	10	(6)	20	(12)	14	(8)	5	(3)	173
**Age**													
≤49	39	(59)	16	(24)	4	(6)	12	(18)	5	(8)	1	(2)	66
50-69	207	(66)	67	(21)	30	(10)	44	(14)	22	(7)	13	(4)	315
≥70	471	(71)	96	(15)	77	(12)	53	(8)	42	(6)	17	(3)	664
**Sex**													
Female	322	(68)	88	(19)	57	(12)	60	(13)	40	(8)	12	(3)	476
Male	395	(69)	91	(16)	54	(10)	49	(9)	29	(5)	19	(3)	569
**Ethnicity**													
White	696	(69)	173	(17)	107	(11)	108	(11)	66	(7)	30	(3)	1013
Asian	9	(56)	3	(19)	1	(6)	1	(6)	1	(6)	0	(0)	16
Black	4	(67)	2	(33)	1	(17)	0	(0)	1	(17)	0	(0)	6
Mixed	3	(75)	1	(25)	0	(0)	0	(0)	0	(0)	1	(25)	4
Other	5	(83)	0	(0)	2	(33)	0	(0)	1	(17)	0	(0)	6
**Socio-economic status (quintiles)**													
1st (Most deprived)	114	(71)	28	(18)	12	(8)	17	(11)	9	(6)	2	(1)	160
2nd	126	(68)	34	(18)	19	(10)	24	(13)	14	(8)	4	(2)	186
3rd	174	(74)	41	(18)	17	(7)	24	(10)	12	(5)	9	(4)	234
4th	132	(63)	39	(19)	29	(14)	19	(9)	13	(6)	9	(4)	210
5th (Least deprived)	171	(67)	37	(15)	34	(13)	25	(10)	21	(8)	7	(3)	255
**Modified Rankin Score 6M**													
0-1 *(no symptoms-no significant difficulties)*	264	(58)	101	(22)	51	(11)	58	(13)	27	(6)	10	(2)	456
2-3 *(slight-moderate disability)*	319	(75)	66	(16)	45	(11)	38	(9)	32	(8)	6	(1)	423

4-5 *(moderate severe-severe disability)*	119	(81)	12	(8)	12	(8)	12	(8)	10	(7)	15	(10)	147
Unknown	15	(79)	0	(0)	3	(16)	1	(5)	0	(0)	0	(0)	19
**Living with**													
Alone	214	(67)	48	(15)	38	(12)	33	(10)	22	(7)	7	(2)	321
Partner	412	(69)	110	(18)	65	(11)	65	(11)	36	(6)	18	(3)	599
Relative/friend	51	(65)	18	(23)	6	(8)	11	(14)	8	(10)	2	(3)	79
In care/hospital	33	(85)	3	(8)	2	(5)	0	(0)	3	(8)	4	(10)	39
Unknown	7	(100)	0	(0)	0	(0)	0	(0)	0	(0)	0	(0)	7
**Getting out and about**													
In their own car	238	(65)	93	(26)	22	(6)	47	(13)	19	(5)	12	(3)	365
Public Transport/Taxi	145	(64)	32	(14)	35	(16)	18	(8)	13	(6)	6	(3)	226
Relative/friend	278	(75)	44	(12)	43	(12)	41	(11)	32	(9)	6	(2)	371
Walk or bike	12	(57)	3	(14)	5	(24)	2	(10)	2	(10)	1	(5)	21
Other	44	(71)	7	(11)	6	(10)	1	(2)	3	(5)	6	(10)	62
**Living situation**													
Own home	632	(68)	164	(18)	105	(11)	100	(11)	59	(6)	25	(3)	937
Relative's home	25	(69)	7	(19)	3	(8)	5	(14)	4	(11)	1	(3)	36
Care-home	29	(91)	2	(6)	2	(6)	0	(0)	4	(13)	5	(16)	32
Supported living	16	(84)	4	(21)	1	(5)	4	(21)	2	(11)	0	(0)	19
Other	15	(71)	2	(10)	0	(0)	0	(0)	0	(0)	0	(0)	21
**Employment**													
Full-time	50	(60)	18	(21)	4	(5)	18	(21)	5	(6)	4	(5)	84
Part-time	31	(66)	12	(26)	6	(13)	8	(17)	4	(9)	0	(0)	47
Retired/Semi-retired	538	(70)	119	(16)	86	(11)	62	(8)	51	(7)	18	(2)	770
Seeking employment	3	(30)	5	(50)	0	(0)	3	(30)	0	(0)	1	(10)	10
Unable to work	87	(71)	24	(20)	13	(11)	17	(14)	8	(7)	6	(5)	122
Other	8	(67)	1	(8)	2	(17)	1	(8)	1	(8)	2	(17)	12
**Post-stroke fatigue**													
Yes	637	(69)	174	(19)	95	(10)	103	(11)	67	(7)	26	(3)	925
No	63	(65)	3	(3)	15	(16)	5	(5)	2	(2)	4	(4)	97
Unknown	17	(74)	2	(9)	1	(4)	1	(4)	0	(0)	1	(4)	23
**Post-stroke depression**													
Yes	410	(71)	96	(17)	64	(11)	74	(13)	51	(9)	21	(4)	575
No	280	(65)	77	(18)	45	(10)	34	(8)	18	(4)	8	(2)	434

Unknown	27	(75)	6	(17)	2	(6)	1	(3)	0	(0)	2	(6)	36
**Post ADL help**													
Yes	334	(76)	48	(11)	45	(10)	38	(9)	34	(8)	19	(4)	438
No	377	(63)	129	(22)	65	(11)	70	(12)	34	(6)	12	(2)	599
Unknown	6	(75)	2	(25)	1	(13)	1	(13)	1	(13)	0	(0)	8
**Post-event important activities**													
Craft, skills, hobby	75	(71)	17	(16)	10	(10)	10	(10)	9	(9)	2	(2)	105
Every day	336	(69)	89	(18)	50	(10)	56	(11)	30	(6)	20	(4)	490
Fitness and exercise	250	(62)	88	(22)	46	(11)	56	(14)	30	(8)	14	(4)	402
Social Group/socialising	173	(68)	47	(19)	39	(15)	31	(12)	15	(6)	9	(4)	254
**Post-event visual impairment**													
Yes	202	(67)	43	(14)	38	(13)	35	(12)	34	(11)	11	(4)	301
No	423	(68)	126	(20)	66	(11)	62	(10)	28	(5)	18	(3)	623
Unknown	92	(76)	10	(8)	7	(6)	12	(10)	7	(6)	2	(2)	121
**Post-event hearing impairment**													
Yes	189	(68)	45	(16)	25	(9)	27	(10)	27	(10)	7	(3)	279
No	437	(68)	119	(19)	75	(12)	66	(10)	34	(5)	20	(3)	639
Unknown	91	(72)	15	(12)	11	(9)	16	(13)	8	(6)	4	(3)	127
**Post-event speech impairment**													
Yes	156	(72)	35	(16)	26	(12)	28	(13)	25	(12)	12	(6)	218
No	461	(68)	124	(18)	71	(10)	72	(11)	36	(5)	18	(3)	680
Unknown	100	(68)	20	(14)	14	(10)	9	(6)	8	(5)	1	(1)	147

Supplementary Information S2. Number of participant characteristics identified within the 'facilitator' themes as a proportion (%) of the total participant characteristics identified for responses to the 'helped' question

Participant characteristics	Family Support (n=286)		Healthcare Support (n=221)		Well-being & fitness (n=183)		Friend Support (n=164)		Self-management (n=152)		Returning to normality (n=70)		'helped' response total (n=820)
**Event**													
Stroke	234	(35)	201	(30)	149	(22)	135	(20)	127	(19)	55	(8)	673
TIA	52	(35)	20	(14)	34	(23)	29	(20)	25	(17)	15	(10)	147
**Age**													
≤49	19	(40)	10	(21)	11	(23)	18	(38)	11	(23)	7	(15)	47
50-69	85	(36)	75	(32)	59	(25)	39	(16)	49	(21)	24	(10)	238
≥70	182	(34)	136	(25)	113	(21)	107	(20)	92	(17)	39	(7)	535
**Sex**													
Male	153	(32)	137	(29)	122	(26)	74	(16)	71	(15)	40	(8)	474
Female	133	(38)	84	(24)	61	(18)	90	(26)	81	(23)	30	(9)	346
**Ethnicity**													
White	279	(35)	214	(27)	177	(22)	161	(20)	149	(19)	68	(9)	794
Asian	2	(20)	1	(10)	1	(10)	0	(0)	2	(20)	1	(10)	10
Black	2	(25)	3	(38)	3	(38)	1	(13)	1	(13)	1	(13)	8
Mixed	1	(50)	1	(50)	1	(50)	1	(50)	0	(0)	0	(0)	2
Other	2	(33)	2	(33)	1	(17)	1	(17)	0	(0)	0	(0)	6
**Socio-economic status (quintiles)**													
1st (Most deprived)	34	(34)	27	(27)	30	(30)	23	(23)	18	(18)	9	(9)	99
2nd	47	(32)	34	(23)	31	(21)	27	(18)	30	(20)	8	(5)	147
3rd	63	(37)	48	(28)	34	(20)	34	(20)	30	(18)	20	(12)	171
4th	76	(37)	52	(26)	47	(23)	34	(17)	32	(16)	15	(7)	203
5th (Least deprived)	66	(33)	60	(30)	41	(21)	46	(23)	42	(21)	18	(9)	200
**Modified Rankin Score (6M)**													
0-1 *(no symptoms-no significant difficulties)*	176	(34)	107	(21)	124	(24)	111	(22)	110	(21)	53	(10)	515

2-3 *(slight-moderate disability)*	80	(34)	81	(34)	47	(20)	43	(18)	39	(16)	15	(6)	239
4-5 *(moderate severe-severe disability)*	29	(49)	31	(53)	10	(17)	9	(15)	2	(3)	2	(3)	59
Unknown	1	(14)	2	(29)	2	(29)	1	(14)	1	(14)	0	(0)	7
**Living with**													
Alone	72	(30)	52	(22)	45	(19)	68	(29)	48	(20)	17	(7)	239
Partner	182	(37)	146	(29)	127	(26)	74	(15)	87	(18)	47	(9)	498
Relative/Friend	29	(41)	16	(23)	10	(14)	19	(27)	17	(24)	6	(9)	71
In care/hospital	2	(22)	6	(67)	0	(0)	2	(22)	0	(0)	0	(0)	9
Unknown	1	(33)	1	(33)	1	(33)	1	(33)	0	(0)	0	(0)	3
**Getting out and about**													
Own car	112	(29)	88	(23)	104	(27)	76	(19)	90	(23)	39	(10)	392
Relative/Friend	109	(48)	77	(34)	43	(19)	44	(20)	23	(10)	14	(6)	225
Public Transport/Taxi	56	(34)	43	(26)	30	(18)	39	(24)	33	(20)	15	(9)	165
Walk/Bike	4	(21)	5	(26)	3	(16)	3	(16)	3	(16)	2	(11)	19
Care home/community	0	(0)	3	(75)	1	(25)	0	(0)	0	(0)	0	(0)	4
Unable	3	(50)	1	(17)	0	(0)	0	(0)	1	(17)	0	(0)	6
Unknown	2	(22)	4	(44)	2	(22)	2	(22)	2	(22)	0	(0)	9
**Living situation**													
Own home	266	(35)	206	(27)	177	(23)	150	(20)	145	(19)	66	(9)	770
Relative's home	15	(60)	5	(20)	4	(16)	9	(36)	3	(12)	4	(16)	25
Care-home	0	(0)	5	(63)	0	(0)	0	(0)	0	(0)	0	(0)	8
Supported living	3	(30)	3	(30)	0	(0)	2	(20)	3	(30)	0	(0)	10
Other	2	(29)	2	(29)	2	(29)	3	(43)	1	(14)	0	(0)	7
**Employment**													
Full-time	34	(37)	25	(27)	21	(23)	25	(27)	22	(24)	13	(14)	92
Part-time	16	(31)	19	(37)	13	(26)	12	(24)	10	(20)	6	(12)	51
Retired/Semi-Retired	209	(34)	151	(25)	134	(22)	115	(19)	111	(18)	48	(8)	613
Seeking employment	2	(40)	2	(40)	2	(40)	1	(20)	1	(20)	1	(20)	5
Unable to work	21	(43)	20	(41)	12	(25)	9	(18)	6	(12)	2	(4)	49
Other	4	(40)	4	(40)	1	(10)	2	(20)	2	(20)	0	(0)	10

**Post-stroke fatigue**													
Yes	246	(36)	189	(28)	142	(21)	142	(21)	125	(19)	52	(8)	676
No	36	(29)	25	(20)	39	(32)	20	(16)	25	(20)	15	(12)	123
Unknown	4	(19)	7	(33)	2	(10)	2	(10)	2	(10)	3	(14)	21
**Post-stroke depression**													
Yes	119	(36)	92	(28)	61	(19)	69	(21)	56	(17)	16	(5)	327
No	158	(34)	121	(26)	118	(25)	90	(19)	93	(20)	50	(11)	467
Unknown	9	(35)	8	(31)	4	(15)	5	(19)	3	(12)	4	(15)	26
**Post ADL Help**													
Yes	106	(41)	90	(35)	46	(18)	47	(18)	26	(10)	13	(5)	257
No	178	(32)	129	(23)	136	(24)	116	(21)	125	(22)	57	(10)	558
Unknown	2	(40)	2	(40)	1	(20)	1	(20)	1	(20)	0	(0)	5
**Post-event important activities**													
Craft and hobby	35	(34)	23	(22)	27	(26)	37	(36)	20	(19)	14	(14)	104
Everyday	181	(46)	128	(33)	83	(21)	96	(25)	82	(21)	44	(11)	390
Fitness and well-being	124	(36)	102	(30)	105	(31)	79	(23)	79	(23)	36	(11)	343
Social activities	92	(42)	71	(32)	39	(18)	67	(31)	55	(25)	20	(9)	219
**Post-event visual impairment**													
Yes	72	(44)	54	(33)	37	(23)	30	(18)	34	(21)	11	(7)	163
No	190	(33)	144	(25)	134	(23)	121	(21)	105	(18)	54	(9)	577
Unknown	24	(30)	23	(29)	12	(15)	13	(16)	13	(16)	5	(6)	80
**Post-event hearing impairment**													
Yes	70	(39)	52	(29)	34	(19)	30	(17)	29	(16)	13	(7)	182
No	190	(34)	148	(27)	136	(24)	120	(22)	108	(19)	52	(9)	559
Unknown	26	(33)	21	(27)	13	(17)	14	(18)	15	(19)	5	(6)	79
**Post-event speech**													
Yes	59	(42)	41	(29)	18	(13)	34	(24)	16	(11)	11	(8)	141
No	203	(34)	151	(26)	148	(25)	116	(20)	117	(20)	55	(9)	590
Unknown	24	(27)	29	(33)	17	(19)	14	(16)	19	(21)	4	(5)	89
